# Skilled health attendants’ knowledge and practice of pain management during labour in health care facilities in Ibadan, Nigeria

**DOI:** 10.18332/ejm/99544

**Published:** 2019-02-07

**Authors:** Beatrice Ohaeri, Gbonjubola Owolabi, Justin Ingwu

**Affiliations:** 1 Department of Nursing, University of Ibadan, Ibadan, Nigeria; 2 Oyo State College of Nursing and Midwifery, Ibadan, Nigeria; 3 Department of Nursing Sciences, Faculty of Health Sciences & Technology, University of Nigeria, Enugu, Nigeria

**Keywords:** knowledge, practice, pain management, nurse-midwives

## Abstract

**INTRODUCTION:**

Skilled health attendants occupy an important position in the management of women’s pain during labour. Their professional goal is to ensure safety and minimum pain in labour. It has been revealed that nurse-midwives are deficient in knowledge and practice of pain management during labour. Hence, this study examined skilled health attendants’ knowledge and practice of pain management in health care facilities in Ibadan, Nigeria.

**METHODS:**

A cross-sectional design was used to collect data from 227 skilled health attendants, in the maternity units of the three purposively selected hospitals for 12 weeks. A structured questionnaire and observational check lists were used for data collection. Data were analyzed using descriptive statistics and significants level was set with p<0.05.

**RESULTS:**

Results on respondents’ level of knowledge revealed that 6% had low knowledge, 40.5% moderate, and 56.8% had a high level. The majority, 79.7%, were registered nurse-midwives (RN/RM) and 90.1% employed reassurance for pain relief. No significant associations were found between respondents’ level of education and reassurance, exercise, allay of fear, use of drugs, and TENS (p>0.05). However, there were significant associations between respondents’ educational level and rubbing of back/massage, position change, cold/warm bath, relaxation, and social support (p<0.05).

**CONCLUSIONS:**

It is recommended that seminars and workshops should be organized regularly and assessment tools should be supplied, to enhance effective pain assessment as this will provide adequate and holistic labour-pain management by nurse-midwives.

## INTRODUCTION

Pain is a universal phenomenon experienced by all persons and pain management is one of the most vital aspects of patient care. Pain as a phenomenon has been defined as an ‘unpleasant sensory and emotional experience associated with actual or potential tissue damage or described in terms of such damage’^1^. On the other hand, ‘pain is whatever the experiencing person says it is, existing wherever he or she says it does’^2^. Labour pain is not an exception, which is practically a product of interactions between physiological and psychological factors^3^. The physiological factors, such as uterine contractions, dilation and effacement are essential parts of labour and are significant contributors to labour pain^3^. Psychological factors such as stress, anxiety, fear, a sense of loss of control and a sense of abandonment contribute to labour pain^3,4^. Pain in labour has been reported by researchers as severe, agonizing and unbearable^5-8^. Experience of labour pain has been found to differ in people and culture; it is greatly influenced by cultural and religious beliefs^9^. Regardless of culture, personality or religious beliefs, labour pain experienced by women could be devastating and has been found to be emotionally laden and psychologically disturbing to the health of women^9^.

Pain management entails a series of activities that encompasses pain assessment as well as pain control and or elimination. Safe and optimal labour pain experience utilizes pharmacological and non-pharmacological interventions in achieving painless labour, and it is explicitly anchored by nurse-midwives. The American Pain Society endorse the phrase: ‘Pain as the fifth vital signs’ to increase the prominence of pain management among healthcare professionals. However, pain researches show that nurse-midwives often assess patients’ pain inaccurately^10-11^.

In developed nations, pain-free labour has become the norm, and the majority of women deliver in hospital, while the story is different in most developing countries like Nigeria where only 35% of women deliver in a hospital. Childbirth in Nigeria has been identified as an unhappy event, and it is less satisfactory because there is an unmet need for pain relief in labour^12-13^.

Poor management of pain in labour could have a negative impact on the outcome of labour. For example, several other researchers having summarized relationships between labour pain management and outcome of labour with the quality of life of women and maternal satisfaction about child birth^14-15^. Given the preceding, there is the necessity for appropriate assessment and management of labour pain to ensure that the experience of women remains positive. Nurse-midwives as members of a multidisciplinary team anchoring pain management are responsible for assessing, treating and monitoring pain in labour. Therefore, they need to be very familiar with contemporary guidelines on pain management^16^. It has been identified that skilled health attendants owe patients a moral, humanitarian, and ethical responsibility to manage and relieve their pain during labour^17^, because effective management of labour pain is regarded as a fundamental human right. It also produces childbirth satisfaction and a favourable birth outcome, which are the aims of practising skilled health attendants in maternity units^18^.

Although pain management is one of the most critical aspects of patient care and it is relevant to all skilled health attendants, there have been few published pain management research studies focusing specifically on those working in maternity or labour wards^19-20^. Several studies have identified that there is a gap in the skilled health attendants’ knowledge about pain generally and this could greatly affect their management of pain across all continuum of care^21-22^. There exists a discrepancy between knowledge and management of pain of women in labour^15^. Therefore, this study assessed the skilled health attendants’ knowledge and practice of pain management during labour in Ibadan, Nigeria.

## METHODS

### Study design

A descriptive cross-sectional survey was used to assess skilled health attendants’ knowledge and practices of labour pain management of women in selected healthcare facilities in Ibadan.

### Setting

Three selected secondary healthcare facilities were used. These included Adeoyo Specialist Maternity Hospital (ASMH), Our Ladies of Apostles Catholic Hospital (OLA), Oluyoro, Oke Offa and Ring Road State Hospital (RRSH) in Ibadan, Oyo State. The selected healthcare facilities are all within the Ibadan metropolis, the capital of Oyo State in southwestern Nigeria. Ibadan is the largest city in the West Africa sub-region, a mega city with 2.6 million people^23^. The hospitals were purposively selected based on strategic placement, proximity to the people, patients, turn out and staff strength of the hospital.

### Sample size

The calculated sample size based on Araoye^24^, a design for calculating the sample size, when the population is less than 100000 is 234.

### Sampling technique

A multistage sampling technique was used, two secondary health facilities were randomly selected from four secondary healthcare facilities in Ibadan, whereas one mission hospital was randomly chosen from the two giving a ratio 2:1. Furthermore, a systematic random sampling technique was used for sample selection of participants from each hospital. The choice was aided by the duty roster of the skilled health attendants at the various hospitals.

### Inclusion criteria

Skilled health attendants who were willing to participate, were available during the period of the study and involved in rotation posting to the selected units.

### Exclusion criteria

Skilled health attendants working in specific special units who were not involved in rotation posting, on annual leave and who were not willing to participate in the study.

### Data collection instrument

A structured questionnaire and observational checklist derived from the extensive literature reviewed were used to obtain information on the sociodemographic characteristics of the respondents, knowledge and practice of pain management of women in labour. The instrument was pretested and had a reliability coefficient of 0.69.

### Procedure for data collection

Seven research assistants were recruited and trained on how to administer the questionnaire and observe the participants during labour. Data collection lasted for 12 weeks (August–October 2016). This allowed ample time for multiple observers to accurately observe respondents on the observational checklist. Hence observer bias was controlled. Furthermore, the practice of pain management in labour among the respondents was observed by the research assistants using the participatory method and outcome rated on observational checklist designed for the study.

### Ethical considerations

Ethical clearance was obtained from the Health Research Committees of the three selected hospitals. Also, permission to conduct the research was obtained from the medical directors, chief nursing officers-in-charge of the hospitals, and administrative heads of the selected units. The questionnaires were administered to the respondents after written informed consent was obtained. Each respondent’s consent was obtained after the objectives of the study, and the rights of the respondents were clarified. Confidentiality of information was assured, and participants were not coerced to participate in the study and free to withdraw at any stage of the study, if they so desired without any negative consequences.

### Data analysis

The data obtained were edited manually, entered into the computer with the aid of the Statistical Package for the Social Sciences (SPSS) version 20. Data analysis was done using descriptive Statistics (frequency, proportions, means and standard deviation to summarize variables). Chi-squared was used to test the significance of the association between variables at a significance level of 0.05.

## RESULTS

Although 234 questionnaires were distributed, only 227 were completed well and retrieved. The sociodemographic characteristics of the respondents revealed that most of the respondents (98.2%) were female; married (75.5%), Christian (83.7%) and of Yoruba origin (89.9%). The mean age was 39.7 ± 9.83 years. The majority (79.7%) were RN/RM, while 0.4% had an MSc. The majority according to rank/cadre of respondents were Nursing Officer II (29.5%), while others (26.0%) were Principal Nursing Officers (Table 1).

**Table 1:** Sociodemographic characteristic of respondents

*Variables*		*Frequency*	*Percentages*
Institution	Ring Road	81	35.7
	Adeoyo	106	46.7
	Oluyoro	40	17.6
Age group (years)	<30	42	18.5
	30-39	72	31.7
	40-49	66	29.1
	≥50	47	20.7
Marital status	Single	47	20.7
	Married	172	75.8
	Divorced	3	1.3
	Widowed	5	2.2
Religion	Christianity	190	83.7
	Islamic	37	16.3
Ethnic Group	Yoruba	204	89.9
	Igbo	13	5.7
	Others	10	4.4
Educational qualification	RN	22	9.7
	RN/RM	181	79.7
	BNSc	16	7
	RN/RM/BSc	7	3.1
	MSc	1	0.4
Rank/cadre	NO I	26	11.5
	NO II	67	29.5
	SNO	21	9.3
	PNO	59	26
	ACNO	21	9.3
	AND	33	14.5

RN: registered nurse, RM: registered midwife, NO: nursing officer, SNO: senior nursing officer, PNO: principal nursing officer, ACNO: assistant chief nursing officer, ADN: assistant director of nursing

An assessment of the participants’ level of knowledge of labour pain management revealed that 2.6% had a low level, 40.5% a moderate level, and 56.8% had a high level. Most (96.9%) of the respondents indicated that pain assessment is the first step in labour management, the majority (98.2%) agreed that they could assess pain and equally considered pain in labour as universal. The majority (75.2%) considered pain relief as an integral determinant of the quality of care provided in labour. Almost all (99.1%) of skilled health attendants were positive on the need to obtain a history of onset of regular rhythmic uterine contractions. Also, most of them 92.4% and 96.9%, agreed to determine the duration of painful regular uterine contractions as well as ascertain the interval between the regular rhythmic uterine contractions, respectively. The majority (79.2%), indicated that to assess the intensity of pain they would place their hands at the fundus of the uterus during contractions.

On the reported methods of managing labour pain, 11 different methods were listed, the most commonly chosen method adopted by the respondents was reassurance (90.3%), followed by use of a drug (53.7%). The lowest in the ranking order was TENS (0.8%) (Table 2). The nurse-midwives were asked about drugs that they used in relieving pain during labour, the ranking order was Pethilofan (20%) first, followed closely by Paracetamol (19.6%), analgesics (17.2%) and Fortwin (16.3%), while Ibuprofen (0.8%) was last. Epidural and Entonox analgesia were not popularly chosen, both at ninth position (each 1.6%).

**Table 2:** Cross tabulation between education and labour pain management

*Methods of managing pain*	*Level of education*					*Total sample N (%)*	*χ^2^*	*p*
	*RN*	*RN/RM/*	*BNSc*	*RN/RM/ BSc*	*MSc Nursing*			
Reassurance	22	158	16	7	1	204 (90.3)	6.50	0.17
Use of drug	95	6	3	1	122	157 (53.7)	7.91	0.10
Rubbing of back and massaging of body	6	24	5	3	1	39 (17.2)	13.82	0.01
Position	6	24	5	3	1	39 (17.2)	13.82	0.01
Cold/warm bathing	5	14	3	3	1	26 (11.5)	20.60	0.00
Relaxation	0	4	3	1	0	8 (3.5)	15.05	0.01
Social support	2	0	1	1	0	4 (1.8)	18.30	0.00
Informing progress of labour	2	2	0	0	0	4 (1.8)	7.71	0.10
Exercise	1	2	0	0	0	3 (1.3)	2.14	0.71
Allay of fear	0	3	0	0	0	3 (1.3)	0.77	0.94
TENS	0	2	0	0	0	2 (0.9)	0.51	0.97

Table 2 also shows the association between educational status of skilled health attendants and methods of pain management of women in labour. The result of the hypothesis showed that there were no significant associations between respondents’ educational level and reassurance (p=0.17), exercise (p=0.71), allay of fear (p=0.94), use of a drug (p=0.10), and TENS physiotherapy (p=0.97). However, there were significant associations between respondents’ educational level and rubbing-of-back/massaging-of-body (p=0.01), position change (p=0.01), cold/warm bathing (p=0.00), relaxation (p=0.01) and social support (p=0.00).

On the practice of pain assessment of women in labour, the skilled health attendants were observed using the checklists. However, on the use of assessment tools revealed a numeric rating scale not being used, the verbal rating scale that appeared popular was also not properly used. On the use of visual analogue scale to assess women in labour pain, none of the respondents from Oluyoro hospital used the scale, while 42.2% and 32.7% from Ring Road specialist hospital and Adeoyo maternity hospital used it, respectively.

On the assessment of labour pain, obtaining a history of onset of painful regular rhythmic uterine contractions was observed to be 100% at Ring Road Speciality Hospital and about 85% at Oluyoro catholic hospital, the lowest. All the respondents at the three hospitals ascertained the interval between the regular rhythmic uterine contractions. On the assessment of the intensity of pain by placing hands on the fundus during contraction, the respondents at Ring Road Speciality Hospital used the examination, and a propotion above average (60%) was observed at Oluyoro catholic hospital practicing the procedure.

## DISCUSSION

In our study, most of the respondents had a good knowledge level, which is in line with the report by Ogboli-Nwasor, in which 94.8% of respondents in their study population agreed that pain relief is needed during labour 9 , and by Ojerinde et al. in which more than two-thirds of the respondents were knowledgeable about management of pain in labour^25^. However, this is unconnected with the quality of training of respondents in the study population. Respondents knowledge about assessment, and the use and availability of pain assessment tools, seems to be at variance with the respondents’ profession, especially compared with the responses from data on the observational checklist in the study settings. Similar observations have been noted by others^26^. Furthermore, some barriers affected the management of labour pain by skilled health attendants in the study settings. Notable among the barriers is the unavailability of assessment tools in the study settings. The majority of respondents understood that labour pain could be managed using non-pharmacological methods and reassurance, which were popularly used by the study participants. Many authors gave similar assertions in their study population^9,21,15,27^. It had been ascertained that skilled health attendants’ level of knowledge could be a contributory factor for the popularity in the use of a non-pharmacological approach to pain management for women in labour^22^. Furthermore, the significant relationship observed between educational qualifications and management of pain using non-pharmacological methods is supported by various authors^28-30^. Specifically, Sahile et al.^31^ stated that professional qualifications were significantly associated with the practice of non-pharmacological labour-pain management methods, and skilled attendants with higher qualifications were 2.87 times more likely to use labour-pain management methods than those who had low level qualifications. Knowledge was seen as an important factor affecting the quality of care and ultimately pain relief and fulfilment in care^25^. It has been stated that the education of skilled attendants can improve the management of labour pain and use of labour pain relief methods^32^.

The popularity of the use of non-pharmacological techniques could breach the gap created by the unavailability of pain relief drugs, and as reported: ‘pain relief drugs are fast disappearing from our labour units’. Furthermore, nurse-midwives active use of non-pharmacological approaches can positively impact on pain management of women in labour^9^. It has been well documented that massage decreases labour pain as well as lessens the dependence on analgesics^33^; also informing mothers about the progress of labour reduces pain^34-35^.

Use of opioids was popular among the participants, and Pethilofan was the drug of choice. This finding is at variance with a study where Pethidine was a popular drug^9^. The majority (70.9%) of respondents gave the drug to relieve pain during labour, which contradicts the findings of Tasnum in which only 6.2% of respondents thought a woman should receive analgesia^36^. Only 1.6% of respondents used epidural analgesia, which is in line with Ogboli-Nwasor who reported that epidural analgesia was not used by any of the providers of pain relief in their study^9^. It is, however, at variance with the observation of Hoger et al. who reported that approximately 60% of women, or 2.4 million each year, choose epidural analgesia for pain relief in labour^37^. It was also reported in Australia that 30% of women who had a normal delivery used epidural analgesia^38^.

Although the skilled health attendants claimed they were knowledgeable about labour pain management, further findings revealed that respondents had inadequate knowledge of and misconceptions about pain relief interventions. Possible reasons include inadequate continuing education on pain management and being unaware of having insufficient knowledge about pain management^39^.

Pain assessment is the first step in pain management, the majority of the respondents (79%) chose wrong assessment tools although respondents claimed to be knowledgeable about labour pain management. This is in line with the observation of some researchers who reported that midwives often assess a patient’s pain inaccurately^10,11^. Inaccurate assessment of pain is seen as problematic because wrong assessment is tantamount to a wrong diagnosis, invariably resulting in either overestimation or underestimation of pain with the resultant inadequate pain management of women in labour^7,8^. Quality care and maternal satisfaction will be then a mirage^7,8^.

In this study, 60–90% of women in labour experienced severe pain at some point during child birth. This observation contradicts the study in which about 68.4% of respondents never had any drug during labour, and only 10.5% of their respondents received pain medication^36^. Similarly, only 23.9% of respondents had drugs to relief pain during labour in the present study. Therefore, skilled health attendants’ previous experience can influence their willingness to offer pain relief drugs to women in labour.

Some myths and misconceptions identified in this study significantly influenced the management of pain during labour. Notable among the study population these were: cultural belief, overestimation or underestimation of pain, no intervention if one expressed pain, it is an extra cost to ask for a pain relief measure, and of course one might end up in an instrument delivery, for example caesarean section, if one expressed pain during labour. These observations are in line with the views of other researchers^2,36,39^.

### Strengths and limitations

In this study, the duty roster of skilled health attendants was used to select the study participants, which eliminated sampling errors. Also, an observational technique was also used to observe how the skilled heath attendants assessed pain during labour, by using the assessment tools.

The limitation of the study was that the participants were nurses and midwives, making it impossible to generalize the findings to other health care professionals.

## CONCLUSIONS

Provision of assessment tools is pivotal to enhancing holistic labour pain management, hence stake holders should be informed on the need to ensure an adequate supply of assessment tools and anaesthetic agents. It is essential for skilled health attendants to be trained on how to use pain management in labour, which should be considered as an important topic of discourse in the Mandatory Continuing Professional Development Programme for nurses and midwives. Labour pain management should be considered as one of the prerequisites for the renewal of a license for midwives. Skilled health attendants’ managers, as advocates, should ensure that awareness is created in the community about bad practices that compel women to endure pain in labour, whereas painless labour experience is the norm globally, especially in developed countries. Drugs like analgesics for relief of pain should be available, accessible and affordable at the three levels of care: primary, secondary and tertiary. Future research should compare labour-pain management at different levels of healthcare delivery systems, including federal and private hospitals.
